# Pathogen-Targeted Clinical Development to Address Unmet Medical Need: Design, Safety, and Efficacy of the ATTACK Trial

**DOI:** 10.1093/cid/ciad097

**Published:** 2023-05-01

**Authors:** Richard R Watkins, Bin Du, Robin Isaacs, David Altarac

**Affiliations:** Division of Infectious Diseases, Department of Medicine, Northeast Ohio Medical University, Rootstown, Ohio, USA; State Key Laboratory of Rare, Complex and Critical Diseases, Medical Intensive Care Unit, Peking Union Medical College Hospital, Beijing, China; Entasis Therapeutics, Waltham, Massachusetts, USA; Entasis Therapeutics, Waltham, Massachusetts, USA

**Keywords:** antimicrobial resistance, antibiotics, sulbactam-durlobactam, ATTACK trial

## Abstract

There is a crucial need for novel antibiotics to stem the tide of antimicrobial resistance, particularly against difficult to treat gram-negative pathogens like *Acinetobacter baumannii-calcoaceticus* complex (ABC). An innovative approach to addressing antimicrobial resistance may be pathogen-targeted development programs. Sulbactam-durlobactam (SUL-DUR) is a β-lactam/β-lactamase inhibitor combination antibiotic that is being developed to specifically target drug-resistant ABC. The development of SUL-DUR culminated with the Acinetobacter Treatment Trial Against Colistin (ATTACK) trial, a global, randomized, active-controlled phase 3 clinical trial that compared SUL-DUR with colistin for treating serious infections due to carbapenem-resistant ABC. SUL-DUR met the primary noninferiority endpoint of 28-day all-cause mortality. Furthermore, SUL-DUR had a favorable safety profile with a statistically significant lower incidence of nephrotoxicity compared with colistin. If approved, SUL-DUR could be an important treatment option for infections caused by ABC, including carbapenem-resistant and multidrug-resistant strains. The development program and the ATTACK trial highlight the potential for pathogen-targeted development programs to address the challenge of antimicrobial resistance.

Drug-resistant bacterial infections caused an estimated 4.95 million deaths worldwide in 2019 [1]. The continued global spread of antimicrobial resistance (AMR) has created an urgent need for novel antibiotics. Unfortunately, antibiotic discovery and development have not kept pace with the rapid evolution of AMR in bacterial pathogens. The problem of AMR is particularly concerning in *Acinetobacter baumanii* due to the limited number of therapeutic options against it [2]. In the United States, investigators found that carbapenem-resistant *A. baumanii* causes approximately 1.2 cases per 100 000 persons, the vast majority of which occur among patients with exposure to a healthcare facility within the preceding year [3]. *A. baumanii* has an extraordinary genetic plasticity that bestows a high capacity to acquire AMR traits including against carbapenems [4]. Among hospitalized patients, it is not uncommon to find multidrug-resistant (MDR; resistance to at least 3 classes of antimicrobials), extensively drug-resistant (XDR; MDR plus resistance to carbapenems), and pan-drug-resistant (XDR plus resistance to polymyxins) *A. baumanii* isolates, thus making them very challenging for clinicians to treat with our current antibiotic armamentarium.

Historically, antimicrobial drug development has focused on discovering broad-spectrum agents that can be used as empiric therapy to treat serious infections [5]. Clinical dogma is that broad-spectrum antibiotics are particularly important early in the course of an infection when the offending pathogen is not yet known. However, this approach encourages overprescribing and inappropriate use, thereby increasing AMR [6]. Broad-spectrum antibiotics have deleterious effects on the host microbiome, particularly in the gastrointestinal tract, causing selection pressure for the development of more resistant bacteria (eg, vancomycin-resistant enterococci and *Clostridiodes difficile*). Expert recommendations have provided guidance on managing resistance, including restricting antibiotic use, antibiotic stewardship programs, improved diagnostic testing to identify causative pathogens, and appropriate use of empiric therapy [7]. Yet, despite these recommendations, increasing AMR remains an ongoing challenge.

## DEVELOPMENT OF PATHOGEN-TARGETED ANTIMICROBIALS

**Figure 1.**
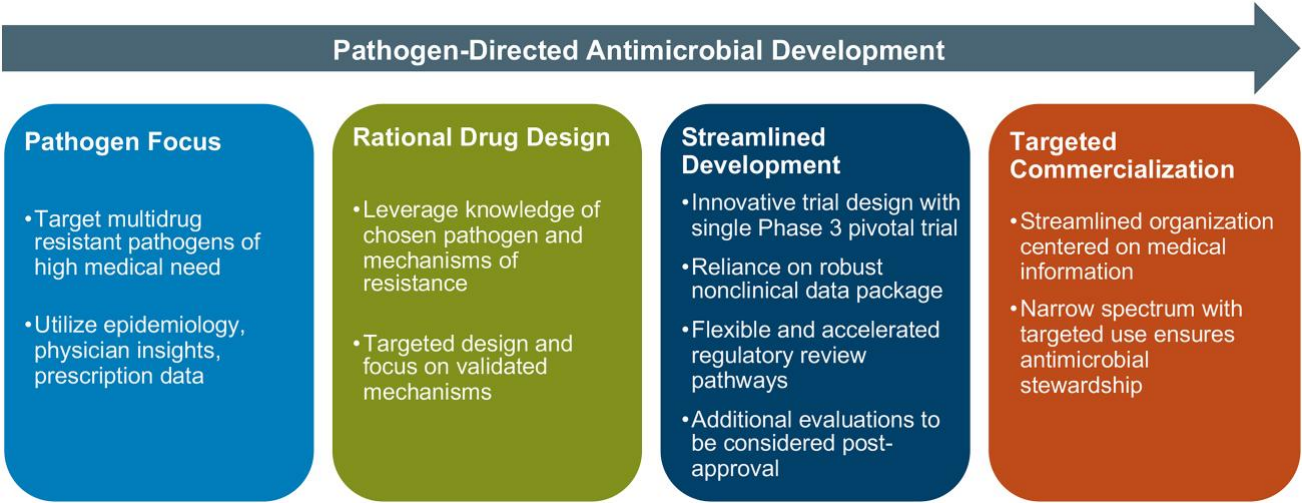
Pathogen-targeted development program (adapted from [8]).

Pathogen-targeted antimicrobial drug development is one tool for addressing the increasing challenge caused by AMR [5, 8, 9] (Figure 1). Alternatively called precision antibiotics, pathogen-targeted antimicrobials are agents that selectively kill a single or a very small number of species, target a specific resistance phenotype, or disrupt a particular pathogenesis mechanism [10]. While it is possible to use pathogen-targeted antimicrobials once routine culture and sensitivities are available, the downside is that the goals of improved antibiotic stewardship and minimizing the emergence of resistance will not be fully actualized due to the continued reliance on first-line broad-spectrum agents. Thus, the need to rapidly identify an infecting pathogen and determine the antibiotic susceptibility is important when narrow-spectrum or pathogen-specific antimicrobials are being considered, especially empirically. As the field of diagnostic testing is rapidly evolving and new ‘omics’ technologies are increasingly able to provide clinicians with information faster and more accurately than traditional testing methods, pathogen-specific therapies may become easier to implement [11]. Additionally, diagnostic testing may improve outcomes as the early administration of appropriate antibiotics has been shown to reduce morbidity and mortality [12].

In 2012, the Infectious Diseases Society of America (IDSA) issued a white paper with recommendations on study designs for clinical trials of antimicrobials for treating drug-resistant pathogens that included suggestions for conducting superiority clinical trials and the use of rapid diagnostic testing to confirm the presence of target pathogens [13]. In recent years, the Food and Drug Administration (FDA) has provided some clarity around the regulatory requirements for developing antimicrobials for treating serious infections that includes recommendations on acceptable, novel study designs, and inclusion of smaller safety databases for approval. For example, the FDA has developed guidance for drug development programs including the Limited Populations Pathway and Antibacterial Therapies for Patients with an Unmet Medical Need for the Treatment of Serious Bacterial Diseases. This initiative and others were designed to streamline the development process for patient populations with the highest medical need [9, 14, 15]. The draft guidance released in 2017 and revised in 2022 highlighted that the FDA has determined that it is appropriate to exercise the broadest flexibility in applying statutory standards while preserving guarantees for safety and efficacy [15]. Specifically, this guidance discusses a more concise development program that includes a single small and statistically rigorous registration trial.

Working with experts, the FDA crafted recommendations and guidance to industry for developing pathogen-targeted antibiotics [9, 14]. The key points were the following: (1) focus on a single pathogen at multiple body sites and organs; (2) optimize the pharmacokinetic (PK)/pharmacodynamic (PD) profile; (3) conduct a single phase 3 noninferiority or superiority clinical trial, with supportive safety data from previous phase 1 and 2 studies; and (4) rapid diagnostic testing to confirm the causative pathogen should be used whenever possible. Using this approach, rationally designed antibiotics can be developed that target MDR pathogens and incorporate a streamlined development and regulatory pathway to approval.

The designs of clinical trials for pathogen-targeted antimicrobials face some unique challenges that are not encountered with those for traditional broad-spectrum agents. Infections due to MDR pathogens in nosocomial settings often arise in the presence of prolonged hospital stays, antibiotic use, indwelling device usage, and complicated illnesses, making them more difficult to treat. Clinical trials to evaluate effective therapies for MDR pathogens are equally difficult because of these factors, along with the challenge of identifying the targeted patient population while facing diagnostic uncertainty and delay [8]. This is further complicated by small patient sample sizes and statistical ambiguity. For example, establishing an appropriate noninferiority margin is challenging when there is limited or varied information on comparator response rates. In the Combating Antibiotic-Resistant Enterobacteriaceae (CARE) Trial, which evaluated plazomicin in patients with bloodstream infections (BSIs) or pneumonia caused by carbapenem-resistant Enterobacteriaceae, only a fraction of the target number of subjects were enrolled [16]. The CARE trial thus highlights the daunting challenges faced by clinical trial investigators focusing on MDR pathogens.

## SULBACTAM-DURLOBACTAM, A PATHOGEN-TARGETED ANTIMICROBIAL

The development of sulbactam-durlobactam (SUL-DUR) is a pathogen-targeted response to infections caused by *Acinetobacter baumanii-calcoaceticus* complex (ABC). The first generation of β-lactamase inhibitor sulbactam has been in clinical use since 1986. Sulbactam inhibits a subset of serine β-lactamases but also binds to penicillin-binding proteins (PBPs) including PBP1a, PBP1b, and PBP3 in ABC, resulting in antibacterial activity in these organisms [17, 18]. However, sulbactam itself is subject to degradation by a broad range of β-lactamases [19]. A novel non–β-lactam diazabicyclooctane (DBO) β-lactamase inhibitor, durlobactam (previously ETX2514), was discovered using structure-based drug design, computational chemistry, and medicinal chemistry. The design hypothesis was based on a combination of increased chemical reactivity, improved enzymatic binding, optimized gram-negative permeation, and physicochemical properties suitable for intravenous dosing [19]. Durlobactam expresses broad-spectrum activity against class A, C, and D β-lactamases [20]. Given the prevalence of class D carbapenemases in ABC, durlobactam is positioned to address carbapenem resistance in these species [20].

A number of reports describe the in vitro antibacterial activity of SUL-DUR against contemporary clinical isolates of ABC [21–26]. The largest of these was a global surveillance study conducted between 2016 and 2021, which showed that durlobactam decreased the maximum inhibitory concentration (MIC) of an antibiotic at which 90% of the isolates are inhibited (MIC_90_) of sulbactam against 5032 ABC from more than 32 µg/mL to 2 µg/mL, with 98.3% of isolates susceptible to 4 µg/mL or less of SUL-DUR, its preliminary breakpoint [22]. In addition to having potent activity in vitro, SUL-DUR was shown to have in vivo efficacy in preclinical animal models of infection [20, 27].

The tolerability and PK of SUL-DUR were also investigated in 6 phase 1 studies in healthy volunteers and in a phase 2 study of hospitalized patients with complicated urinary tract infection or acute pyelonephritis [28–32]. SUL-DUR demonstrated a consistent and predictable PK and tolerability profile that was similar in both healthy subjects and hospitalized patients, with excellent penetration into pulmonary tissues [29]. Furthermore, SUL-DUR was well tolerated in these phase 1 and phase 2 studies [28–32]. The Acinetobacter Treatment Trial Against Colistin (ATTACK) trial (ClinicalTrials.gov: NCT03894046) was a global, randomized, active-controlled phase 3 noninferiority trial that evaluated the safety and efficacy of SUL-DUR compared with colistin in patients with serious infections from carbapenem-resistant ABC (CRAB) [33]. The design of this phase 3 trial was in accordance with the FDA guidance for industry on antibacterial therapies for patients with an unmet medical need for the treatment of serious bacterial diseases [9]. The primary endpoint was all-cause mortality at 28 days. There were 207 patients recruited from 95 clinical sites in 17 countries. All patients had to have an infection caused by ABC. The trial was conducted in 2 parts: part A was a randomized, comparative study that evaluated SUL-DUR versus colistin in patients with hospital-acquired bacterial pneumonia, ventilator-associated bacterial pneumonia, or BSIs, and part B was an open-label study that included SUL-DUR for patients with infections caused by ABC strains with resistance to colistin or polymyxin B, or patients who had otherwise failed colistin or polymyxin B therapy. All patients received imipenem/cilastin background therapy in parts A and B to ensure coverage of possible polymicrobial infections. Part A was the primary safety and efficacy analysis population and part B provided additional safety and supportive efficacy data in patients with *Acinetobacter* infections that may have been ineligible for part A.

SUL-DUR met the primary efficacy endpoint of noninferiority for 28-day all-cause mortality in the primary analysis population (part A) and CRAB microbiologically modified intention-to-treat population (n = 125). Mortality in the SUL-DUR group was 19.0% (12/63) versus 32.2% (20/62) in the colistin group (treatment difference: −13.2%; 95% confidence interval: −30.0%, 3.5%; noninferiority margin: 20%). In all study populations, similar trends favoring SUL-DUR were observed in 14-day and 28-day all-cause mortality. Clinical cure rates at Test of Cure were 61.9% for SUL-DUR and 40.3% for colistin. Furthermore, the 28-day all-cause mortality in part B was 17.9%, consistent with that observed in part A.

The primary safety objective of the ATTACK trial was a comparison of the incidence of nephrotoxicity as measured by the Risk, Injury, Failure, Loss, and End-Stage Kidney (RIFLE) classification in part A [34]. SUL-DUR achieved the primary safety objective with a statistically significant reduction in nephrotoxicity (13.2%, 12/91) compared with colistin (37.6%, 32/85; *P* = .0002). Overall adverse events (AEs) were comparable between the treatment groups, with 87.9% (80/91) in the SUL-DUR recipients and 94.2% (81/86) in the colistin recipients in part A and 89.3% (25/28) in part B. Drug-related AEs occurred in 12.1% (11/91) in the SUL-DUR group versus 30.2% (26/86) in the colistin group in part A and in 10.7% (3/28) in part B.

ATTACK was the first randomized controlled trial to evaluate an investigational antibiotic against a specific drug-resistant gram-negative pathogen. SUL-DUR is the first investigational drug to demonstrate efficacy in a 28-day all-cause mortality trial focused on CRAB. These positive results have facilitated the continued development of SUL-DUR towards the ultimate goal of global regulatory approval.

## CONCLUSIONS

The successful progression of SUL-DUR for the treatment of *A. baumannii* infections demonstrates the potential for pathogen-focused antibacterial development in the fight against drug-resistant infections. SUL-DUR is a unique β-lactam/non–β-lactam DBO β-lactamase inhibitor combination antibiotic that offers a novel approach to overcoming β-lactam resistance in ABC. The ATTACK trial was a pivotal study that explored the efficacy and safety of SUL-DUR in patients with severe infections due to CRAB. If approved, SUL-DUR will be an important treatment option for patients with serious and life-threatening infections caused by *A. baumanii*, including carbapenem-resistant strains.

## Notes

***Acknowledgments***. The authors acknowledge the editorial assistance of Richard S. Perry, PharmD, which was supported by Entasis Therapeutics, Waltham, MA.

***Financial support*.** This work was sponsored by Entasis Therapeutics, a wholly owned subsidiary of Innoviva, Inc. The authors did not receive any fees for authorship.

***Supplement sponsorship*.** This article appears as part of the supplement “Sulbactam-durlobactam, a Targeted β-lactam/β-lactamase Inhibitor, for MDR *Acinetobacter*,” sponsored by Entasis Therapeutics Inc., a wholly owned subsidiary of Innoviva, Inc.
